# Development of a Green Polymeric Membrane for Sodium Diclofenac Removal from Aqueous Solutions

**DOI:** 10.3390/membranes13070662

**Published:** 2023-07-12

**Authors:** Camila Suliani Raota, Janaina da Silva Crespo, Camila Baldasso, Marcelo Giovanela

**Affiliations:** Área do Conhecimento de Ciências Exatas e Engenharias, Universidade de Caxias do Sul, Rua Franscisco Getúlio Vargas, 1130, Caxias do Sul 95070-560, Brazil

**Keywords:** poly(vinyl alcohol), citric acid, crosslinking, statistical analysis, physicochemical characterization

## Abstract

Water-soluble polymers provide an alternative to organic solvent requirements in membrane manufacture, aiming at accomplishing the Green Chemistry principles. Poly(vinyl alcohol) (PVA) is a biodegradable and non-toxic polymer renowned for its solubility in water. However, PVA is little explored in membrane processes due to its hydrophilicity, which reduces its stability and performance. Crosslinking procedures through an esterification reaction with carboxylic acids can address this concern. For this, experimental design methodology and statistical analysis were employed to achieve the optimal crosslinking conditions of PVA with citric acid as a crosslinker, aiming at the best permeate production and sodium diclofenac (DCF) removal from water. The membranes were produced following an experimental design and characterized using multiple techniques to understand the effect of crosslinking on the membrane performance. Characterization and filtration results demonstrated that crosslinking regulates the membranes’ properties, and the optimized conditions (crosslinking at 110 °C for 110 min) produced a membrane able to remove 44% DCF from water with a permeate production of 2.2 L m^−2^ h^−1^ at 3 bar, comparable to commercial loose nanofiltration membranes. This study contributes to a more profound knowledge of green membranes to make water treatment a sustainable practice in the near future.

## 1. Introduction

The increasing demand for water to accommodate population and economic growth has spoiled water resources [1]. The presence of contamination in waters has been causing environmental damage and affecting human health [2]. Among these water contaminants, emergent organic micropollutants (e.g., pharmaceuticals, personal care products, and endocrine disruptors) represent an additional challenge since they can be harmful at trace levels and are not removed by traditional water treatment systems [2,3]. The potential consequences have motivated governmental organizations to create regulations, such as the European Union Watch List [4] and the Contaminant Candidate List from the United States Environmental Protection Agency [5], which aim to monitor substances of concern when present in water for human consumption.

In this sense, advanced water treatments have been developed to meet water quality parameters, such as advanced oxidation processes [6,7,8], adsorption [9], and membrane separations [2,10]. Of these methods, membrane technology has the most potential due to its efficiency in removing organic micropollutants, low energy consumption, small equipment size, affordable initial cost, and no requirements for the addition of chemicals [1,10,11,12,13]. The specific membrane process is selected according to the target micropollutant. Due to the smaller pores, nanofiltration (NF) or reverse osmosis (RO) membranes are the most frequently used to treat water contaminated with organic micropollutants [3,14]. The removal rates vary, usually above 80% when using RO and tight NF membranes [2,15] but inferior (30–60%) when using a loose NF membrane [10]. For instance, the removal of diclofenac (a non-steroid anti-inflammatory drug with a high global incidence in waters [16,17]) is above 90% using BW30 (RO, Dow Filmtec, DuPont, Wilmington, DE, USA) [18], NF90 (NF, Dow Filmtec) [18], NF270 (NF, Dow Filmtec) [19,20], HL (NF, GE Osmonics, Minnetonka, MN, USA) [20], AFC 30, and 40 (NF, PCI Membrane Systems, Poland) [21], and less than 50% using the membranes NF10 and 50 (NF, Hydranautics, Oceanside, CA, USA) [22].

The global membrane technology market was estimated at USD 24.6 billion in 2022 [23], which is in enormous contrast to that in developing regions, such as South America, which corresponded to only USD 1.6 billion [24]. In this geographic area, Brazil is leading the demand for membrane technologies due to the recent changes in legislation aiming for improvement in the performance of existing wastewater treatment systems [24]. The membrane process efficiency relies on the operational conditions, the physicochemical properties of the pollutant (charge, molecular weight, and polarity), and membrane characteristics (pore size, charge, hydrophilicity, functional groups on the surface, and morphology) [2,14,25]. The need for better membrane performance, especially regarding the higher permeate production and reduced operational pressure, has been boosting the research on new membranes [26]. In general, rejection mechanisms seem to rule the performance of NF and RO membranes: exclusion by size, electrostatic repulsion, and adsorption are reported as the most relevant processes [2,10,14]. This can be achieved by exploring the polymer for membrane preparation, modification of the fabrication process, and use of additives or nanoparticles, thereby creating unique properties and characteristics of pore size, hydrophilicity, charge, and fouling resistance, among others [1,10,11,15,27,28]. Moreover, society’s increasing concern for sustainability motivates the development of greener membranes [13].

Sustainability is referred to by the “Brundtland Report” of the United Nations (1987) as a development that accomplishes the needs of present generations without compromising future generations [29]. This concept goes with the Green Chemistry principles established by Anastas and Warner in 1998 [30], which aimed to protect people and the planet by reducing waste, conserving energy, and discovering alternatives to harmful substances [31,32,33]. Membrane technologies are considered a green process [32]. However, membrane fabrication should be reconsidered to minimize toxicity and environmental impact [33,34]. The drawbacks are the use of petroleum-based and non-biodegradable polymers and the massive need for organic solvents, which are usually toxic and harmful to humans and the environment [27,33,34]. Alternatives are green solvents (e.g., ionic liquids or water) and reagents derived from renewable sources, such as natural and/or biodegradable polymers [32,35,36,37].

Reports of the development and application of green membranes in the literature are still scarce but have gained visibility in the last 10 years [32]. The preparation of membranes using green solvents, such as Rhodiasolv^®^ Polarclean and Cyrene™, is more frequently observed. Polarclean^®^ is derived from an industrial process by-product (2-methylglutaronitrile), which is usually burned as waste. It is biodegradable, not toxic or mutagenic, and water-miscible [38]. Cyrene™ comes from cellulose and is water soluble, with low toxicity, a high boiling point, and low molecular weight. It has physicochemical properties comparable to those of *N*-methyl-2-pyrrolidone and dimethylformamide [39]. Water has also been used in membrane preparation, even though its use is limited to water-soluble polymers [34]. These green solvents were used in the fabrication of polyethersulfone [38,39,40], poly(vinylidene fluoride) [39,41], polysulfone [40], cellulose acetate [40], and poly(vinyl chloride) [42] membranes, from microfiltration to nanofiltration and even membrane distillation, with competitive performances when compared to state-of-the-art membranes.

More recently, the use of greener polymers combined with less toxic additives has been reported. Zhang et al. (2020) [43] prepared electrospun polylactide membranes with gallic acid and titanium oxide for oil–water separations. Electrospun cellulose acetate nanofibers were produced by Oldal et al. (2023) [44] using green solvents (dimethyl carbonate and cyclopentanone) and additives (tetrabutylammonium bromide and sophorolipid-based biosurfactants from honey yeast), and the authors highlighted its biodegradability. PVA-based membranes were prepared for water desalination by pervaporation and purification of emulsified oil wastewater containing Pb(II) ions. Salt rejection higher than 99.9% was obtained from a silica-PVA membrane (silica extracted from rice husk) [45], and a hydrogel made of PVA and chitosan showed 99.9% oil rejection combined with 97.4% metallic ion removal [46]. An innovative gradient crosslinked PVA membrane was prepared by Zeng et al. (2023) using a diphthalic anhydride, producing an excellent combination of permeate flux (88.6 L m^−2^ h^−1^) and salt rejection (88.7%, Na_2_SO_4_) [47]. Indeed, PVA water solubility, among other properties, makes it an alluring choice for green polymeric membrane fabrication [48].

PVA is a synthetic semi-crystalline polymer obtained from the hydrolysis of poly(vinyl acetate), in which hydroxyl groups replace original ester groups totally or partially, generating PVA with varied degrees of hydrolysis [48,49,50]. Some properties depend on the degree of hydrolysis, such as water solubility and crystallinity. This is due to the effect of residual ester groups on the strong intermolecular bonds formed between hydroxyl groups [48,49,50]. The characteristics of biodegradability, biocompatibility, non-toxicity, and fiber- and film-forming ability make PVA a strong candidate for green membrane fabrication [46,48,49]. As a membrane, PVA has satisfactory mechanical and thermal properties but excellent chemical resistance, besides its easy processing, flexibility, transparency, and anti-fouling characteristics [48,49,50,51]. PVA-based membranes are employed in direct methanol fuel cells, biosensors, gas separation, pervaporation, polymer electrolyte membranes, and water and wastewater treatment, especially NF and RO membranes [48,49,52]. When applied in aqueous environments, PVA must have its water solubility controlled through crosslinking methods [47,53].

Crosslinking is a simple and established method for bonding polymer chains to form a crosslinked network [28,50,54]. For PVA, crosslinking occurs on the hydroxyl groups from the polymer backbone, thus reducing the number of available sites to interact with water [28,55]. The restrictions imposed on the mobility of the polymer chain affect the crystallinity, swelling, and solubility, besides thermal, chemical, and mechanical properties [28,49,50,53,55,56]. Specifically, controlling physical and chemical crosslinking methods can tune the membrane properties [49,53,57]. Physical crosslinking relies on crystallinity changes, physical interactions, and molecular entanglements, usually triggered via freeze–thaw cycles, heat treatment, and γ-irradiation exposure [49,53,58,59,60]. Chemical crosslinking, on the other hand, is the most popular method due to its simplicity and the myriad of crosslinkers available. A crosslinker is a chemical compound with reactive groups (at least two) that form covalent bonds with the hydroxyl groups of PVA chains [53,54]. Some examples of crosslinkers are aldehydes, isocyanates, anhydrides, acids, and carboxylic acids [28,49,50,53,54,55,56]. Among these compounds, toxicity, cytotoxicity, carcinogenicity, unpleasant odor, and adverse effects on the membrane (such as promoting nonbiodegradability) are drawbacks, especially for the most employed substance, glutaraldehyde [28,49,53].

**Figure 1:**
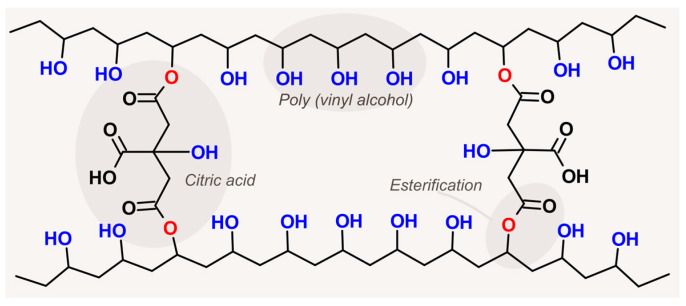
Crosslinked PVA network through an esterification reaction with citric acid. PVA and citric acid structures, besides the reaction sites where the esterification reaction occurs, are highlighted in the grey areas.

For this reason, the scientific community has been exploring more sustainable options for PVA crosslinking, such as carboxyl acids which are considered green crosslinkers due to the properties of non-toxicity, biodegradability, and availability [28]. Accordingly, citric, sulfosuccinic, maleic, and succinic acids have received increased attention [28,47,55]. Indeed, citric acid is one of the most promising crosslinkers due to its eco-friendly production (via fermentation), availability, and inexpensive cost [61]. Overall, the use of carboxylic acids for PVA crosslinking involves three steps: (i) mixture with PVA in aqueous solution, (ii) casting or fiber preparation, and (iii) final crosslinking using heat treatment or microwave irradiation. The final crosslinking facilitates the esterification reaction between PVA and carboxyl acid [28], forming a network as illustrated in Figure 1. The extent of the crosslinking reaction impacts the membrane performance (e.g., permeate flux and rejection) since it controls porosity, pore size, swelling, structural stiffness, and compactness [28,52]. Therefore, optimum crosslinking should be studied and determined for each membrane application.

In view of all these aspects, the present work had the following research questions: (i) How do the membrane’s chemical, physical, and morphological properties vary with the time and temperature of the crosslinking? (ii) What are the factors (namely time and temperature) or combinations of factors that significatively influence membrane crosslink, based on its performance (permeate flux and sodium diclofenac (DCF) removal)? (iii) What are the best conditions of time and temperature to crosslink a PVA membrane with citric acid aiming at the best performance? Aiming to answer these research questions, the design of experiments (DOE) methodology and statistical analysis were employed to investigate the crosslinking reaction aiming at the optimum conditions that combine permeate flux and DCF removal from aqueous solutions. Furthermore, the membranes produced according to the DOE were extensively characterized to understand the effect of crosslinking on membrane performance.

## 2. Materials and Methods

### 2.1. Poly(Vinyl Alcohol)-Based Membrane Preparation

The green membranes were prepared by the evaporation of an aqueous solution containing the polymer (PVA), crosslinker (citric acid), and additives (glycerol and silver nanoparticles, AgNPs). Preliminary experiments optimized the concentration of each component, and the composition with good film formation properties was 8% (*w*/*v*) of PVA, 10% (*w*/*w_PVA_*) of citric acid, 20% (*v*/*v*) of AgNPs solution, and 4% (*v*/*v*) of glycerol. It should be noted that no further catalyst was used, and the crosslinking was completed with a thermal treatment [28,62]. The incorporation of additives aimed to modify the characteristics of the crosslinked PVA membrane to obtain better performance (in the case of AgNPs) and facilitate its processing (in the case of glycerol). Green synthesized AgNPs have a negative net charge (−48 mV, as characterized in previous studies [63]), so its addition is intended to make the membrane surface negative and promote a more efficient removal of negatively charged pharmaceutical compounds, such as DCF [2,3,10,14]. Glycerol was incorporated due to its plasticizer properties [64], aiming to avoid the brittle characteristic of crosslinked PVA [47], which could cause membrane fracture.

PVA powder (hydrolysis > 99%, MW 85,000–124,000 kDa, Sigma–Aldrich, São Paulo, Brazil) was pre-solubilized in distilled water (model Q341-210, Quimis Aparelhos Científicos, Diadema, Brazil) to achieve a concentration of 10% (*w*/*v*). For this, 10 g of PVA was dissolved in 100 mL of distilled water under magnetic stirring (model ARE, Velp Scientifica, Usmate Velate, Italy) for 24 h in a double jacket flask with a constant temperature of 80 °C (model U2C, VEB MLW, Leipzig, Germany). AgNPs were synthesized following the protocol of previous works [63]. Briefly, equal volumes of AgNO_3_ (2.5 mol L^−1^ aqueous solution, ≥99%, Merck, Pinheiros, Brazil) and a hydroalcoholic (50.0% *v*/*v* ethanol-water, Vetec Química Fina Ltd., Duque de Caxias, Brazil) extract of grape pomace (50 g L^−1^, pH = 10.0) were combined, producing AgNPs with an average diameter of 2.9 nm. The complete characterization of the AgNPs can be found in the report from Raota et al. (2019) [63].

The membrane precursor solution preparation consisted of adding 0.32 g of anhydrous citric acid (2-hydroxypropane-1,2,3-tricarboxylic acid, ≥99.5%, Cinética Ltd., Itapevi, Brazil) and 1.6 g of glycerol (propane-1,2,3-triol, ≥99.5%, Vetec Química Fina Ltd., Brazil) to 32 mL of pre-dissolved PVA 10% (*w*/*v*). After mixing for 30 min at 50 °C, 8.0 mL of AgNPs solution was incorporated. Subsequently, air bubbles were removed in an ultrasonic bath (30 min, model USC-1400A, Unique, Indaiatuba, Brazil), and the solution was poured and spread on a glass sheet with a glass blade (wet film of ~1.0 mm). The spread solution dried at room temperature (23 ± 2 °C) for approximately 24 h on a perfectly level surface. The dried membranes were then transferred from the glass sheets to polytetrafluoroethylene sheets and crosslinked in an oven (model AGSEDT, DeLeo, Porto Alegre, Brazil), following the conditions of time and temperature determined by the DOE (described in Section 2.2). After the crosslinking procedure, the membranes were stored at room temperature (23 ± 2 °C) for further experiments.

### 2.2. Optimization of Crosslinking Conditions Using DOE and Statistical Analyses

The parameter optimization for the membrane crosslink occurred through a central composite rotational design (CCRD). The choice of time and temperature factors was based on their relevance to the crosslinking reaction, in addition to the potential combined effect [65]. The central point (condition ‘0,0′) corresponded to 60 min at 130 °C, being pre-optimized parameters by a 2^3^ experimental design performed in previous works [66]. The experimental error evaluation was made by repeating the central point conditions three times [67]. The axial points factors were varied to higher (+1) and lower values (−1), corresponding to 30 and 90 min for time and 120 and 140 °C for temperature. Additionally, four additional points at a distance α = √2 from the central point enabled the analysis by response surface methodology (RSM) [67]. Table 1 shows the CCRD experiments executed in this work, as well as the code for each membrane, following the acronym “M” + “time” + “temperature”. The optimized membrane M110_110 is also mentioned in Table 1, and its determination is discussed in Section 3.5.

Each membrane (codified in Table 1) was evaluated by its DCF rejection and permeate flux production in filtration experiments (described in more detail in Section 2.3). The results obtained in the filtration experiments of the 11 membranes were statistically analyzed using the software Statistica 10. The statistical analysis comprised the following: (i) identification of outliers by analyses of variation coefficient, standard error, symmetry, and kurtosis, (ii) verification of assumptions of normality using Shapiro–Wilk and Hartley tests for subsequent variance analysis, (iii) variance analysis using ANOVA to verify significant effects of factors and their interactions, (iv) determination of the mathematic model to generate the response surfaces, and (v) identification of optimal values of factors by response surface analysis and desirability function [67].

### 2.3. Evaluation and Characterization Techniques

Ultraviolet-visible (UV-Vis) and Fourier-transform infrared (FTIR) spectroscopies, differential scanning calorimetry (DSC), the swelling ratio in water, water contact angle (WCA), and scanning electron microscopy (SEM) revealed the physical and chemical characteristics of the membranes. The performance evaluation of the membranes described in Section 2.3.4 was made regarding rejection and permeate flux through filtration experiments.

#### 2.3.1. Spectroscopic Characterization

The ultraviolet-visible light adsorption of the membranes was evaluated in a spectrophotometer (DU530, Beckman, Indianapolis, IN, USA) between 200 and 690 nm, with a 1.0 nm resolution. The membranes were pinned on the sample holder and positioned in the light beam. Duplicate spectra were collected, and the reported result is the average spectrum. FTIR analyses were executed in an infrared spectrometer with an attenuated total reflection (ATR) accessory (Nicolet iS10, Thermo Scientific, Waltham, MA, USA). Samples were stored in a desiccator for 24 h and then measured between 4000 and 400 cm^−1^ (128 scans, resolution 2.0 cm^−1^, transmittance mode).

#### 2.3.2. Thermal Characterization

DSC analyses (DSC-60, Shimadzu, Japan) used about 10 mg of each sample in aluminum pans. The analysis used a heating and cooling rate of 10 °C min^−1^ from room temperature (~23 ± 2 °C) to 225 °C under a nitrogen atmosphere (50 mL min^−1^). Thermal data for the enthalpy of fusion ($\Delta H_{f}$) and glass transition (*T_g_*) and fusion (*T_f_*) temperatures were extracted from the first run, while the temperature of crystallization (*T_c_*) from the cooling run; crystallinity (*X_c_*) was calculated using Equation (1) [68]:

*X_c_* (%) = (Δ*H_f_*/Δ*H*_*f*,100%_) × 100 (1)

where Δ*H_f_* is the variation of fusion enthalpy of the sample (J g^−1^) normalized by its content in the membrane and Δ*H_f,100%_* is the theoretical fusion enthalpy corresponding to PVA hypothetically 100% crystalline (162 J g^−1^) [69].

#### 2.3.3. Physical and Morphological Characterization

Membrane swelling in water was evaluated regarding mass (*S_M_*) and dimension (*S_D_*) using Equations (2) and (3) [70], respectively:

*S_M_* (%) = [(*M_w_* − *M_d_*)/*M_d_*] × 100 (2)

*S_D_* (%) = [(*A_w_* − *A_d_*)/*A_d_*] × 100 (3)

where $M_{w}$ is the wet mass (g), $M_{d}$ is the dry mass (g), $A_{w}$ is the wet area (mm^2^), and $A_{d}$ is the dry area (mm^2^). Squares of 4.0 cm^2^ of each sample (six replicates) were dried in a desiccator for 24 h (23 ± 2 °C). The sample dry dimensions and weight were measured with a digital caliper (0–150 mm, Digimess, São Paulo, Brazil) and a precision scale (AD500, Marte, São Paulo, Brazil). After this procedure, the samples were hydrated for 24 h in beckers with 30 mL of distilled water (temperature of 23 ± 2 °C) [71], and the wet dimensions and weights were measured.

Water contact angle analysis was evaluated via the sessile drop method by disposing 100 μL of distilled water on the membrane attached to a flat surface. Images were collected immediately after the drop deposition (digital camera, DMC-FZ40, Panasonic, Kadoma, Japan) and analyzed with the software Surftens 3.0. WCA measurements were determined by the average of three drops on three replicates of each membrane [72]. Surface and cross-section images of the membrane were obtained with SEM (MIRA3, Tescan, Brno, Czech Republic). Sample preparation was done via cryogenic fracture (for cross-section images), followed by attachment to a stub with carbon tape and gold sputtering (2 min, Desk V, Denton Vacuum, Moorestown, NJ, USA). SEM images magnified 10,000 and 2000 times were collected using an acceleration voltage of 12.0 kV with the secondary electrons’ detector.

#### 2.3.4. Filtration Experiments

Filtration experiments were performed in a system composed of a feed tank, pump, membrane holder, manometer, valves, and tubes, as shown in Figure S1. The filtration occurred in crossflow mode with the feed stream entering tangentially to the membrane holder, under batch conditions (concentrate stream returning to the feed tank). The membrane had an active filtration area of 12.6 cm^2^ and was pre-conditioned in distilled water for 30 min before placing it in the membrane holder. Soaking the membrane in water aimed to stabilize the membrane swelling before the filtration and remove any possible unreacted PVA chains. Distilled water or a DCF (≥98%, Sigma–Aldrich, São Paulo, Brazil) aqueous solution at 0.01 g L^−1^ [18,73,74] was the feed, kept at a constant temperature of 22 ± 2 °C (analogic thermometer −10 °C/50 °C, Incoterm, Porto Alegre, Brazil). The experimental procedure for filtration experiments consisted of (i) membrane compaction at 6 bar until constant permeate flux during 1 h, (ii) hydraulic permeability with distilled water (decreasing pressure 6–0 bar), (iii) hydraulic permeability with DCF solution (decreasing pressure 6–0 bar), (iv) rejection to DCF (at 6 bar, unless otherwise mentioned), and (v) hydraulic permeability with distilled water after the rejection experiment (decreasing pressure 6–0 bar).

The permeate flux (*J_P_*, L m^−2^ h^−1^) was calculated using Equation (4), where *V* is the permeate volume (L), *A* is the membrane active filtration area (m^2^), and *t* is the time interval of permeate collection (h).

*J_P_* = *V*/(*A* × *t*) (4)

The hydraulic permeability (*L_P_*, L m^−2^ h^−1^ bar^−1^) correlates the permeate flux with the transmembrane pressure (Δ*P*, bar), as shown in Equation (5):

*L_P_* = *J_P_*/Δ*P* (5)

The membrane selectivity was determined by the membrane rejection (*R*) to an analyte (here, DCF) using Equation (6), where *C_F_* (g L^−1^) is the analyte concentration in the feed stream and *C_P_* (g L^−1^) is the analyte concentration in the permeate.

*R* (%) = [(*C_F_* − *C_P_*)/*C_F_*] × 100 (6)

DCF quantification was done through the equation obtained by linear regression of a calibration curve with six standards (0.0003–0.01 g L^−1^, in distilled water) analyzed by ultraviolet-visible spectroscopy (DU530, Beckman, Indianapolis, IN, USA). The samples were measured at 278 nm [75] inside a quartz cuvette with an optic pathway of 1.0 cm. The linear regression was done using the software Origin 9.0, and the equation that represents the calibration curve was y = 32.35837x + 0.00052633, with R^2^ = 0.99994.

## 3. Results and Discussion

The synthesized green membranes were translucid with the characteristic yellowish color of AgNPs [63], irrespective of the crosslinking conditions (see the membrane M60_130 as an example in Figure S2). Pure PVA is translucid and colorless [76]. All the membranes were moderately rigid when dry and flexible when hydrated. The characterization and filtration results, followed by the statistical analyses, are presented in the next sections. For a better comparison and discussion of the results, the characterization of the crosslinked membrane in the optimized conditions (M110_110) was also approached, although its choice was only discussed in Section 3.5.

### 3.1. Spectroscopic Characterization Using Ultraviolet-Visible and Fourier-Transform Infrared Spectroscopies

**Figure 2:**
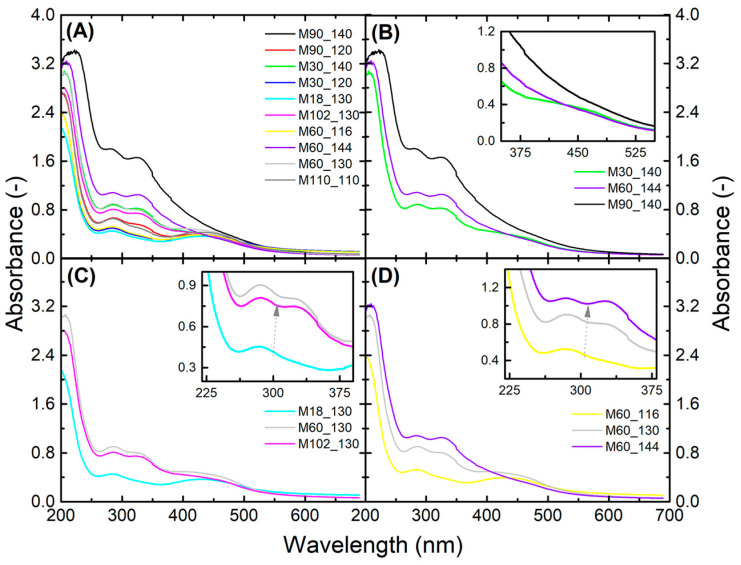
UV-Vis spectra of the membranes crosslinked according to the CCRD. The graph in (**A**) shows all the membranes of the CCRD and the optimized membrane (M110_110), while (**B**) reveals the membranes crosslinked at higher temperatures (140 and 144 °C), (**C**) a comparison of different times at 130 °C, and (**D**) varied temperatures for 60 min. The grey arrows in (**C**,**D**) represent the increase in absorbance with time and temperature, respectively.

The membranes were optically characterized using UV-Vis spectroscopy to disclose possible modifications caused by the crosslinking conditions. The resulting full spectra are shown in Figure 2. It is possible to observe the four most significative bands (Figure 2A) with maxima at around 200–220, 285, 330, and 450 nm. The band 370–530 nm refers to the surface plasmon resonance band of spherical AgNPs [63,77]. This band (370–530 nm) could not be seen in the spectra of membranes crosslinked at higher temperatures during an extended time (M90_140 and M60_144) (Figure 2B, inset), probably masked by the increase in the overall transmittance of the membrane, which Yang et al. (2021) associated to greater crosslink density [78]. The absorption band at around 200 nm may be from residual acetate groups [79] (present in low concentrations even in high hydrolysis degree PVA), which may have merged with the carboxylic acid’s (from citric acid) band at ~209 nm [80]. This band increased intensity and shifted its maximum absorbance from 200 nm (M18_130) to 222 nm (M90_140) (Figure 2A), which can be related to the enhanced linkage of citric acid to the membrane [80] caused by the crosslinking reaction (see Figure S3A for a comparison between pure PVA and PVA with citric acid). Sau et al. (2021) did not observe this band shift in PVA films after heat treatment (no use of crosslinkers) [65]. The bands at around 285 and 330 nm are related to the transitions π→π* and n→π* transitions caused by the resonating carbonyl group [65,79], which had increased intensity with more intense crosslinking conditions of time and temperature (Figure 2C,D). In mild combinations (i.e., reduced time or lower temperature), the band at around 330 nm was not present. Sau et al. (2021) observed an increase in these two bands with time and temperature up to 140 °C and 40 min, associating this with crosslinking due to hydrogen bond formation. Beyond these conditions, film degradation begins, characterized by a greater overall absorbance [65].

**Figure 3:**
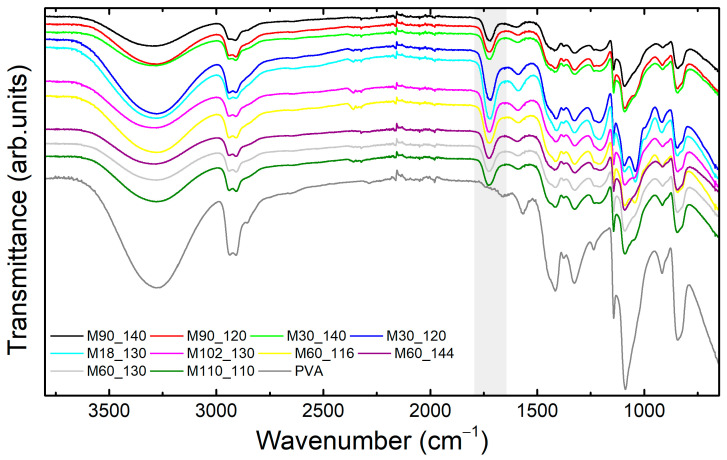
ATR-FTIR spectra of the membranes crosslinked according to the CCRD and the optimized membrane (M110_110) and pure PVA for comparison. Highlighted in grey is the region of C=O stretching of esters (1720 cm^−1^).

FTIR results can give a better comprehension of chemical modifications (Figure 3). In general, bands attributed to the PVA structure could be identified, namely O–H (stretching at 3275 cm^−1^ and bending at 1417 cm^−1^), C–H and C–H_2_ (stretching at 2940 cm^−1^ and 2905 cm^−1^, out-of-plane twisting at 840 cm^−1^), and C–O (bending at 1324 cm^−1^ and stretching at 1088 cm^−1^) [51,65,77]. The intense O–H band occurs due to intramolecular hydrogen bond interactions [65]. Residual acetate bands were not observed (C–O and C=O stretching vibrations at ~1020 cm^−1^ and ~1700 cm^−1^) [65,77].

**Figure 4:**
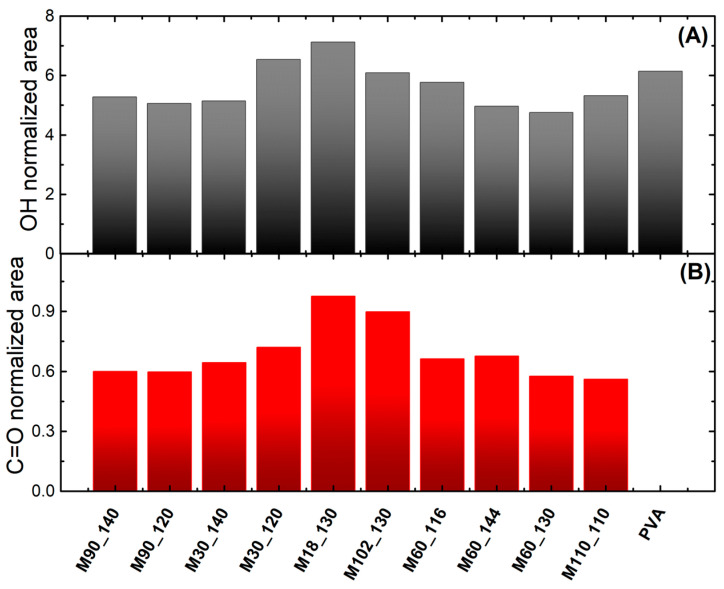
FTIR normalized areas of (**A**) O–H and (**B**) C=O spectra bands. The band areas were normalized by the CH and CH_2_ band areas (2940 cm^−1^ and 2905 cm^−1^). Note: pure PVA had no visible C=O band (~1720 cm^−1^).

The crosslinks formed through the esterification of PVA with citric acid can be observed by the stretching band C=O of esters at 1720 cm^−1^ [54], highlighted by the grey area in Figure 3. This band may also relate to free carboxylic acid groups from unreacted citric acid [77]. The esterification of glycerol by citric acid is also possible to have occurred [81,82]; however, the absence of bands (2226 cm^−1^, 2115 cm^−1^, 1505 cm^−1^, and 1057 cm^−1^ [81]) referred to as the product of the reaction suggests its minor incidence. A reduction of the O–H band (3275 cm^−1^) was expected due to its consumption in the esterification reaction [77,78,83]. In this sense, the analysis through normalized areas (Figure 4) can show more accurately the modifications on O–H and C=O bands since it compensates for the natural variability of intensities caused by the ATR accessory.

The membranes in Figure 4A with lower O–H normalized area than PVA indicate consumption of hydroxyl groups promoted by the esterification reaction [77,78,83]. However, a higher normalized area (as observed for M30_120 and M18_130) may also imply that more citric acid molecules were added to the polymeric structure. Once the amount of citric acid used in all membranes was the same, the differences among the normalized areas of C=O in Figure 4B can be attributed to the esterification degree [77], which was the case of membranes M18_130 and M102_130. Even though FTIR analyses could identify the esterification reaction, the results could not explain the performance of all membranes evaluated. Then, the membranes were investigated using thermal analyses.

### 3.2. Thermal Characterization Using Differential Scanning Calorimetry

**Figure 5:**
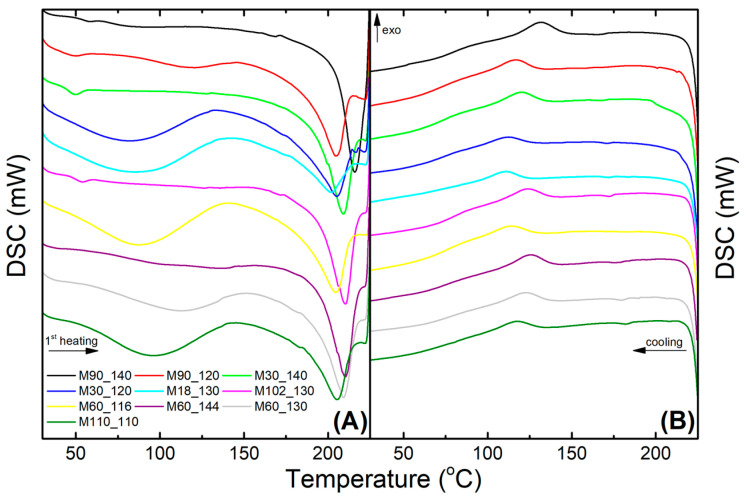
DSC curves of the membranes crosslinked according to the CCRD and the optimized membrane (M110_110): (**A**) first heating run; (**B**) cooling run.

The effects of crosslinking on the thermal characteristics of membranes can be seen in the DSC curves from the heating and cooling run in Figure 5. Crosslinking conditions strongly influenced the membranes’ thermal properties, evaluated at the first heating to not mask the effects of time and temperature of crosslinking. Table 2 shows the main thermal events. *T_g_* values varied from 42.1 to 62.6 °C, while the *T_g_* for pure PVA is reported from 74.0 to 86.0 °C [84,85]. The overall reduction in the *T_g_* of the membranes can be associated with the plasticizing effect of glycerol. As a plasticizer, glycerol reduces the cohesive attraction forces of the polymer chain, facilitating its mobility [71]. Additionally, the reaction of hydroxyl groups with citric acid reduced the intermolecular interactions through hydrogen bonds [86], providing more freedom to the unanchored polymeric chains. Among the membranes, the crosslinking temperature tended to reduce *T_g_* due to the inhibited chain movement caused by crosslinks [71]. A similar effect was also observed in mild temperatures combined with extended time (e.g., M110_110).

*T_f_* values reduced a little in comparison to the reported for pure PVA in the literature (*T_f_* = 224–230 °C [71,84]). As well as *T_g_*, the decrease of PVA chain interactions due to crosslinking caused the reduction of *T_f_* [71]. The diverse crosslinking conditions promoted slight modifications of the membranes’ *T_f_*, ranging from 201.7 °C (M18_130) to 215.9 °C (M90_120). Nataraj et al. (2020) [83] suggest that more crosslinked reactions can increase the molecular weight of PVA, thus requiring higher temperatures to melt. Indeed, the calculated crystallinity goes along with the *T_f_*. The crystallization temperature was lower than the one reported for pure PVA (141–183 °C [78,84]), varying from 110.6 °C (M18_130) to 131.5 °C (M90_120). The reduced *T_c_* is attributed to the inhibited chain mobility caused by the crosslinks, compromising the crystallization [78]. Indeed, the conditions that promoted higher crosslinking (such as higher temperature or the combination of mild temperatures with extended time) gave lower *T_c_* values.

Strong intermolecular interactions from PVA hydroxyl groups are responsible for its semi-crystalline characteristics [65]. This is totally dependent on the hydrolysis degree since residual acetate groups inhibit crystallization [86]. For the membranes (Figure 5A), Δ*H_f_* was normalized by the final PVA content in the membrane (95.9%), and the crystallinity was calculated using Equation (1) (considering the fusion enthalpy of hypothetically 100% crystalline PVA = 162 J g^−1^ [69]). It was observed that higher crosslinking temperatures increased crystallinity (e.g., M30_140, *X_c_* = 51.6%) as well as mild temperatures for an extended time (e.g., M90_120, *X_c_* = 51.6%). Shi et al. (2015) observed the same trend but with the crystallinity determined by X-ray diffraction analyses [87]. Zeng et al. (2023) observed a reduction in crystallinity on highly crosslinked samples and attributed it to the decrease of available sites for hydrogen bonding [47]. It should be noted that in the case of a crosslinked PVA, Δ*H_f_* refers to the melting enthalpy of both the crystalline and crosslinked regions; it is not possible to differentiate them [88]. Overall, DSC results showed that the crosslinking had pronounced effects on the polymer chain mobility, modifying membrane thermal behavior. Another indirect manner to evaluate the extent of crosslinking is to measure the amount of water incorporated by the membrane via swelling measurements.

### 3.3. Physical Characterization via Swelling and Water Contact Angle Analyses

The crosslinking of PVA with carboxylic acids (such as citric acid) creates nanometric voids that can absorb large amounts of water [28], causing membrane swelling. Massic ($S_{M}$) and dimensional swelling ($S_{D}$) were calculated using Equations (3) and (4), respectively, and the results are in Table 3. Swelling values varied with the diverse crosslinking conditions: massic from 23.9 to 41.8% and dimensional from 23.8 to 39.6%. For both cases, M30_120 showed the least swelling and M102_130 the highest. The variations seem to be smooth when compared to other materials, for example, PVA with citric acid for food packaging (but without crosslinking treatment) that achieved almost 400% swelling [62] or pure PVA that completely dissolves in water [62]. The esterification reaction is responsible for keeping the PVA chains anchored and reducing the interstitial volume [89], decreasing the number of water molecules trapped [62], and, consequently, reducing swelling. Sabzi et al. (2020) also observed a reduction in the swelling of PVA hydrogels crosslinked with citric acid when AgNPs were incorporated, attributing the presence of nanoparticles to additional crosslink points [77].

The longer the time or the higher the crosslinking temperature, the greater the swelling (both massic and dimensional). This behavior is counterintuitive, as it was expected that the more crosslinked membranes (promoted by extended time or higher temperatures) would swell less. Jiang et al. (2023) suggested that the presence of non-uniform regions of crosslinks can create areas of loose polymer chains responsible for the swelling [90]. Indeed, when comparing the crystallinity shown in Table 2 with the swelling from Table 3, it is observed that the membrane M102_130 has the highest swelling (*S_M_* = 41.8 ± 6.0%; *S_D_* = 39.6 ± 1.5%) as well as the highest crystallinity (*X_c_* = 50.1%), i.e., poor crosslinking among the chains. Conversely, M30_120 has a similar crystallinity (*X_c_* = 49.7%) but the lowest swelling (*S_M_* = 23.9 ± 3.4%; *S_D_* = 25.5 ± 2.6%). The more relevant difference between those two membranes is the *T_c_* (M30_120 = 111.7 °C; M102_130 = 123.3 °C), which indicates enhanced chain mobility for M102_130, partially explaining the swelling behavior. Those observations suggest that the swelling on crosslinked PVA membranes is a complex mixture of factors, such as crosslinking, crystallinity, and general chain mobility. Harland and Peppas (1989) also observed that the swelling of PVA crosslinked membranes was affected by the crosslinking conditions and the degree of crystallinity [91]. Swelling studies evidence the interaction of the membrane with the solvent (here, water) in all the extensions of the material and provide enough time to reach equilibrium. The wettability, in contrast, reveals the instantaneous effects of water deposited on the membrane surface.

**Figure 6:**
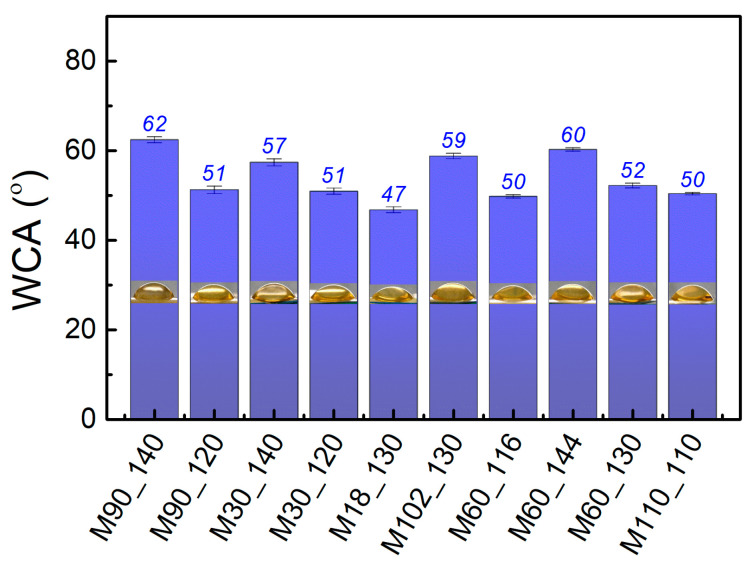
Water contact angle of the membranes crosslinked according to the CCRD and the optimized membrane (M110_110). WCA is reported as the average of the replicates and their standard deviation.

The wettability of the membranes was evaluated through WCA measurements, and the results are shown in Figure 6. PVA is naturally hydrophilic due to the abundance of hydroxyl groups in its structure [78,92]. The crosslinking reaction with citric acid reduced PVA affinity for water without turning the membrane hydrophobic (WCA > 90°) [62]. Indeed, the maximum WCA was 62° for the membrane M90_140. A reduction of hydrophilicity (higher WCA) with elevated temperatures (especially > 140 °C) was observed, as well as the combination of mild temperatures with extended crosslinking time (e.g., M102_130).

The PVA and citric acid crosslinked microfiber mats produced by Yu et al. (2021) varied from 32 to 42° when the citric acid content was increased from 3 to 12%. This was related to the formation of ester bonds between the polymer and citric acid [62]. All of the samples were thermally treated for 8 min at 130 °C, which is comparable with the membrane M18_130 with a WCA of 47°. The WCA results showed diverse water affinity behavior on the surface from the bulk (swelling studies, Table 3). While the crosslinks in the bulk membrane seemed heterogeneous (leading to an anomalous swelling behavior), the surface properties agree with previous results and the literature. Overall, the crosslinking conditions promoted diverse characteristics of the membranes. As relevant as the understanding of the crosslinking reaction is the impact of it on the membrane performance in filtration experiments, discussed in the next section.

### 3.4. Membrane Performance in Filtration Experiments and Statistical Analysis

The results of DCF rejection and permeate flux (at 6 bar) of the membranes prepared according to the CCRD are presented in Table 4. It should be noted that the membranes M90_140 and M30_190 could not have their rejection values calculated since the permeate volume produced was not enough to be analyzed by UV-Vis.

**Figure 7:**
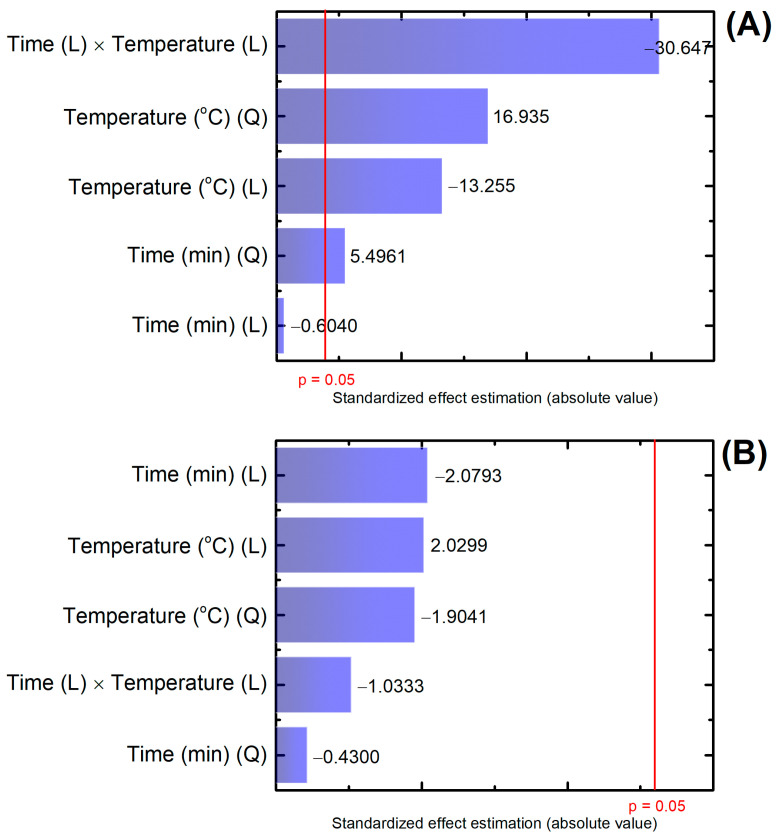
Pareto charts generated by the variance analyses using ANOVA (software Statistica 10) for (**A**) permeate flux and (**B**) DCF rejection. Results of statistical significance considering the confidence interval of 95% (*p* = 0.05). Note: (L) = linear factor, (Q) = quadratic factor.

Based on the results from Table 4, ANOVA analysis was performed using only first- and second-order interactions (linear and quadratic) since they showed effects superior to the other interaction orders [67]. Membranes M90_140 and M30_140 were not considered in the statistical analysis because the DCF rejection could not be determined. Based on the confidence interval of 95% (*p*-value = 0.05), the factors temperature (linear and quadratic), time (quadratic), and the interaction of time and temperature (both linear) had statistical significance on the permeate flux (Table S1). Regarding the rejection, none of the factors showed statistical significance. The effect intensity of the variable on permeate flux and DCF rejection can be visualized on the Pareto charts in Figure 7.

**Figure 8:**
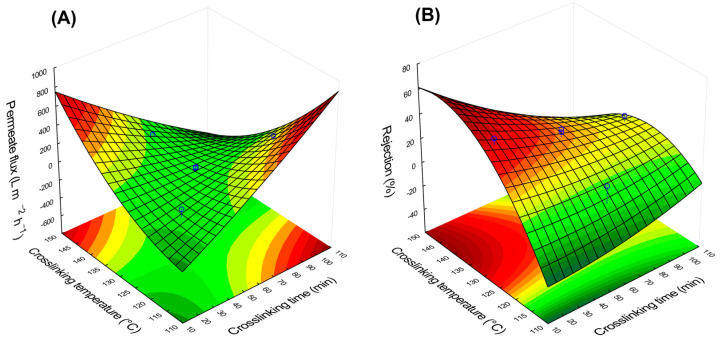
Response surfaces obtained using RSM from the software Statistica 10 for the effect of crosslinking temperature and time on the (**A**) permeate flux and (**B**) DCF rejection. Note: the blue circles refer to the original data.

The interaction of time and temperature (linear) had the most influence on the permeate flux. Between those, the temperature seems to have contributed more. Zeng et al. (2023) observed a negative impact on permeate flux with higher crosslinking temperatures, explained by the reduction of available hydroxyl groups to interact with water on highly crosslinked PVA [47]. The rejection was not significantly affected by any factor. However, the standardized effect estimation of individual factors was similar and slightly more relevant than their combinations. The interaction of factors and their influence on the membrane performance can be better visualized through the RSM graphs (Figure 8).

Figure 8A shows two areas where the combination of time and temperature of crosslinking promotes higher permeate flux: (i) higher temperature with lower time and (ii) reduced temperature with extended time. The easy pass of water through the membrane can be associated with its hydrophilicity, as observed in the FTIR analyses by the excess of hydroxyl groups (Section 3.1) and the reduced WCA (Section 3.3). For the rejection, one region points out the best performance (Figure 8B): higher temperature combined with short crosslinking time. Even though the factors were not statistically significant, a tendency for DCF rejection can be observed. Similar behavior was reported by Medhat Bojnourd and Pakizeh (2018) [93]. They evaluated crosslinked PVA thin films optimized by experimental design and statistical analysis. Higher temperatures tended to increase crystallinity (Section 3.2) and promote more crosslinks between PVA chains (Section 3.3), leading to improved rejection at the cost of reducing the permeate flux. Aiming to achieve the best balance between rejection and permeate production, the desirability function from the software Statistica 10 can be employed to point out the most favorable conditions.

### 3.5. Desirability Function

**Figure 9:**
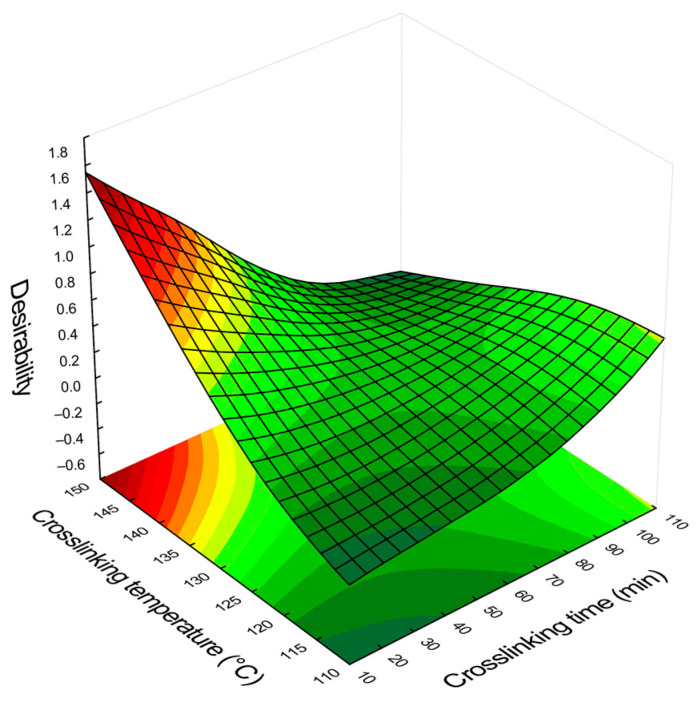
Response surface obtained using the function desirability of the software Statistica 10, combining the optimal values of permeate flux and DCF rejection as a function of the crosslinking parameters (time and temperature).

The blend of the two surfaces of Figure 8 indicates regions where both parameters (permeate flux and rejection) can have their optimal values. This surface is achieved using the desirability function in the software Statistica 10. The surface generated by the desirability function (Figure 9) shows that the crosslinking conditions for the best permeate flux and rejection are at elevated temperature and short crosslinking time (150 °C for 10 min, called M10_150).

**Figure 10:**
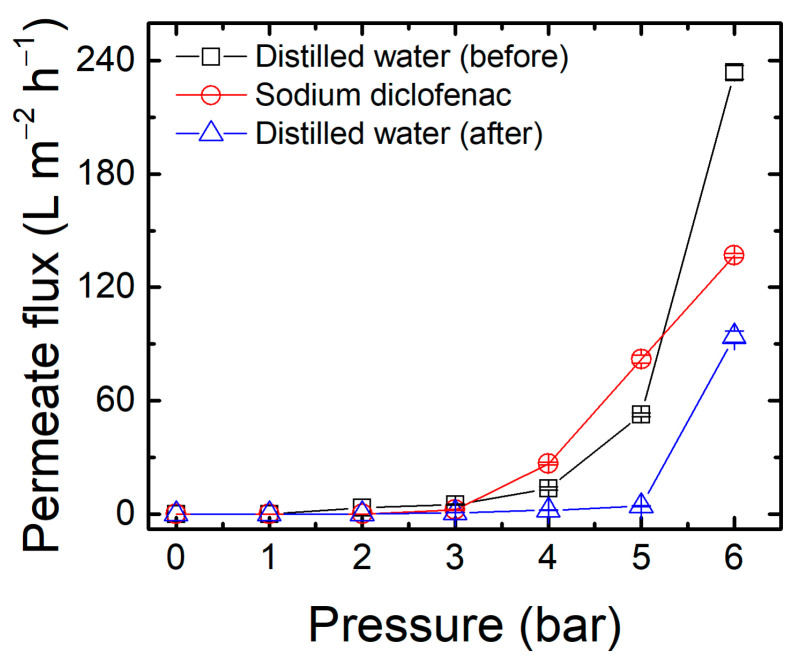
Permeate flux produced by the optimized membrane M110_110 when filtrating a DCF solution (0.01 g L^−1^) and distilled water (before and after filtration with the solution) as a function of the operational pressure.

The new membrane M10_150 was crosslinked in the optimized conditions and tested with filtration experiments to evaluate its performance, and the results showed unsatisfactory DCF rejection (15.3 ± 3.7%). The review by Bolto et al. (2009) [52] on the crosslink of PVA membranes indicates that high temperatures produce unsaturation and scissions in the polymeric chain, which could have compromised its performance. Sau et al. (2021) [65] also observed the “burning” of PVA films at temperatures higher than 140 °C. Figure S3B presents the UV-Vis spectra of membrane M10_150, where it is possible to observe an overall increase in the membrane absorption, which could indicate unsaturations [52]. For this reason, the region with the second highest desirability score in Figure 9 was selected since it has milder temperature conditions for the crosslinking: 110 °C for 110 min (M110_110). This membrane showed satisfactory results, and Figure 10 presents the permeate flux with DCF solution (0.01 g L^−1^) and distilled water (before and after filtration with the analyte solution).

The permeate flux when filtering distilled water increased with the pressure, showing an inflection at 3 bar. This behavior is similar to membranes employed in membrane distillation processes, in which a minimum transmembrane pressure must be applied to allow a liquid to penetrate and transpose the membrane [94,95]. Indeed, PVA has been employed in membrane distillation and pervaporation processes, especially as an active layer in composite membranes [52,96]. In this sense, the non-linear response of permeate flux with pressure may be related to the intrinsic membrane characteristics of PVA, i.e., pore tortuosity, surface tension, contact angle, porosity, and surface roughness [97]. The permeate flux at 3 bar for distilled water was 4.9 L m^−2^ h^−1^, in agreement with that reported by Ahmad et al. (2012) using PVA membranes crosslinked with glutaraldehyde (4.9 L m^−2^ h^−1^, 2.5 bar) [98], and Sakarkar et al. (2020) with a poly(vinylidene fluoride) support membrane coated with PVA crosslinked with glutaraldehyde (1.96 L m^−2^ h^−1^, 3 bar) [99]. The relatively low permeate flux can be related to a dense membrane, as observed in SEM images (Figure S4). The non-linear behavior of permeate flux as a function of pressure was also observed for the DCF solution, however, with lower permeate flux when compared to distilled water (2.2 L m^−2^ h^−1^ at 3 bar, a reduction of 55%). Maryam et al. (2020) affirm that the interaction between DCF and the polymer can cause the adsorption of pharmaceuticals on the membrane surface, reducing the permeate flux [22]. The adsorption also affected the permeate flux of distilled water after the DCF solution (0.72 L m^−2^ h^−1^ at 3 bar), showing an 85% reduction when compared to the first permeation with distilled water.

The rejection mechanism of DCF is supposed to be mainly due to the Donnan effect [22] since DCF is negatively charged at its natural pH (~5.8), and the green synthesized AgNPs [63] conferred a negative charge to the membrane. Using the M110_110 membrane, DCF rejection started at ~90% in the first minutes of permeation and then stabilized at ~44% after 60 min. This behavior raises the hypothesis of pharmaceutical adsorption on the membrane surface, which stabilized after saturation. In comparison with the performance of DCF removal reported using commercial membranes (Table 5), the membrane M110_110 has a similar performance to the loose nanofiltration membrane NF50 [22], with the advantages of lower pressure (3 bar) and higher permeate flux (2.2 L m^−2^ h^−1^).

## 4. Conclusions

The present work presented the optimization of crosslinking conditions for a green PVA-based membrane using a DOE (central composite rotational design) and a statistical evaluation of the results obtained from filtration experiments (permeate flux and DCF removal). Characterization analyses showed that elevated crosslinking temperature caused damage to the polymer that spoiled membrane performance and lower temperatures did not promote enough crosslinks through esterification. On the other hand, a mild temperature during an extended time demonstrated a good balance for the esterification reaction, resulting in a good permeate flux (consuming PVA hydroxyl groups but not reducing hydrophilicity) and adequate selectivity towards DCF (tightening the membrane network but not increasing the membrane crystallinity). The statistical analysis revealed that the permeate flux was influenced by the temperature (linear and quadratic factors) besides the interaction between time and temperature of crosslinking. Regarding the DCF rejection, none of the factors had statistical significance. Based on the surface generated by the desirability function from Statistica 10 software, the best combination of permeate flux and DCF removal occurred at the crosslinking conditions of 110 min at 110 °C. The green membrane crosslinked at these conditions showed a permeate flux of 2.2 L m^−2^ h^−1^ at 3 bar with a DCF removal of 44%, comparable to a loose nanofiltration commercial membrane but with the advantage of lower pressure and higher permeate production. Finally, the results showed the synthesis of a green membrane with performance comparable to commercial membranes, but only using sustainable reagents in mild preparation conditions. The developed green membrane has the potential to be employed on a large scale; however, further studies regarding its stability and durability in long-run filtration processes are still required.

## Figures and Tables

**Table 1:** Composite central rotational design of experiments performed in this work to optimize the crosslinking reaction of green membranes.

Membranes ^1^	Factor Codes ^2^		Time (min)	Temperature (°C)
	x_1_	x_2_		
M90_140	+1	+1	90	140
M90_120	+1	−1	90	120
M30_140	−1	+1	30	140
M30_120	−1	−1	30	120
M18_130	−√2	0	18	130
M102_130	+√2	0	102	130
M60_116	0	−√2	60	116
M60_144	0	+√2	60	144
M60_130 #1	0	0	60	130
M60_130 #2	0	0	60	130
M60_130 #3	0	0	60	130
M110_110 ^3^	-	-	110	110

^1^ Membranes named following the acronyms “M” + “time” + “temperature”; #1, #2, and #3 refer to replicates. ^2^ x_1_ = factor 1 (time) and x_2_ = factor 2 (temperature). ^3^ Membrane with the optimized crosslinking conditions (described in Section 3.5).

**Table 2:** Thermal events were obtained from the DSC curves for membranes crosslinked according to the CCRD in addition to the optimized membrane (M110_110).

Membranes	*T_g_* (°C)	*T_f_* (°C)	Δ*H_f_* (J g^−1^)	*X_c_* (%)	*T_c_* (°C)
M90_140	42.1	204.4	75.5	46.4	116.5
M90_120	53.7	215.9	83.9	51.6	131.5
M30_140	44.6	208.9	83.9	51.6	119.5
M30_120	45.8	205.2	80.7	49.7	111.7
M18_130	46.2	201.7	56.4	34.7	110.6
M102_130	49.6	210.4	81.5	50.1	123.3
M60_116	45.1	204.4	75.4	46.3	113.3
M60_144	62.6	210.3	75.3	46.3	125.0
M60_130	60.9	209.2	75.2	46.2	121.8
M110_110	53.9	205.5	55.6	34.2	116.7

**Table 3:** Massic (*S_M_*) and dimensional (*S_D_*) swelling of the membranes crosslinked according to the CCRD as well as the optimized membrane (M110_110). The swelling is reported as the average of the replicates with the standard deviation.

Membranes	*S_M_* (%)	*S_D_* (%)
M90_140	29.9 ± 5.5	36.3 ± 3.2
M90_120	29.6 ± 4.2	37.4 ± 1.3
M30_140	30.0 ± 1.9	38.3 ± 1.7
M30_120	23.9 ± 3.4	25.5 ± 2.6
M18_130	28.9 ± 1.8	23.8 ± 1.1
M102_130	41.8 ± 6.0	39.6 ± 1.5
M60_116	31.9 ± 3.8	36.7 ± 1.5
M60_144	33.4 ± 3.1	38.8 ± 2.3
M60_130	33.0 ± 3.1	38.7 ± 2.6
M110_110	35.2 ± 3.6	46.5 ± 4.5

**Table 4:** Permeate flux (at 6 bar) and DCF rejection of the membranes crosslinked according to the CCRD.

Membranes	Permeate Flux (L m^−2^ h^−1^)	Rejection (%)
M90_140	0.95	-
M90_120	363	0.8
M30_140	1.19	-
M30_120	24.0	3.7
M18_130	9.1	49.3
M102_130	3.6	21.3
M60_116	68.0	11.0
M60_144	73.6	20.7
M60_130 #1	14.2	33.3
M60_130 #2	22.8	15.7
M60_130 #3	10.2	30.8

**Table 5:** Performance of commercial membranes reported in the literature regarding DCF removal from aqueous solutions.

Membranes	MWCO ^1^ (Da)	DCF (g L^−1^)	*R* ^2^ (%)	Pressure (bar)	Permeate Flux/Hydraulic Permeability	Ref.
AFC 30 (PCI Membranes)	100–150	0.02	99.2	25–30	6.04 L m^−2^ h^−1^ bar^−1^	[21]
AFC 40 (PCI Membranes)	200–400	0.02	99.4	15–20	7.11 L m^−2^ h^−1^ bar^−1^	[21]
BW30 (Dow FilmTech)	≈100	0.01	98	20	-	[18]
DL (GE Osmonics)	~150–300	0.16	94	10	3.2 L m^−2^ h^−1^ bar^−1^	[100]
HL (GE Osmonics)	150–300	1.0	90	10	-	[20]
		0.16	99	10	9.5 L m^−2^ h^−1^ bar^−1^	[100]
NF10 (Hydranautics)	3000	0.1	9.7	8	0.003 L m^−2^ h^−1^	[22]
NF50 (Hydranautics)	1000	0.1	43.3	8	0.0007 L m^−2^ h^−1^	[22]
NF90 (Dow FilmTech)	200–400	0.01	98	20	-	[18]
		0.16	98	10	8.7–11.3 L m^−2^ h^−1^ bar^−1^	[100]
NF270 (Dow FilmTech)	200–400	0.001	91	6.9	-	[19]
		1.0	100	10	-	[20]
		0.16	92	10	13.5–18.5 L m^−2^ h^−1^ bar^−1^	[100]
NFX (Synder Filtration)	~150–300	0.16	~100	10	4.2–5.5 L m^−2^ h^−1^ bar^−1^	[100]
TS40 (Trisep Corp)	~200	0.16	99	10	4.2 L m^−2^ h^−1^ bar^−1^	[100]
TS80 (Trisep Corp)	100–200	0.06	~100	5	36 L m^−2^ h^−1^	[101]
		0.16	99	10	4.2 L m^−2^ h^−1^ bar^−1^	[100]
PVA + citric acid, AgNPs, and glycerol	-	0.01	44	3	2.2 L m^−2^ h^−1^	This work

^1^ MWCO = molecular weight cut-off. ^2^ *R* = rejection.

## Data Availability

All data generated or analyzed during this study are included in this published article.

## Supplementary Materials

The following supporting information can be downloaded at: https://www.mdpi.com/article/10.3390/membranes13070662/s1. Figure S1: Graphical illustration of the filtration system: (A) feed container, (B) pump, (C) membrane holder, (D) permeate container, (E) manometer, (F) flow valve. In detail, a photograph of the membrane holder operated in crossflow mode. Figure S2: Photograph of the membrane M60_130. In detail, the translucid characteristic of the membrane. Figure S3: UV-Vis spectra of (A) films made of pure PVA and PVA with citric acid, all submitted to a heat treatment of 110 °C for 110 min, and (B) the comparison of spectra of M10_150 and M110_110. Figure S4: SEM images of the membrane M110_110 from the surface (right) and cross-section (left). The red arrows point to impurities on the membrane surface, while the measurement in yellow refers to the membrane thickness. Table S1: ANOVA results for permeate flux and DCF rejection obtained using the software Statistica 10. Note: F = F-statistics, *p* = *p*-value. In red are the results of statistical significance, with 95% confidence (*p* = 0.05).
