# Supplementary material for: Development of a Green Polymeric Membrane for Sodium Diclofenac Removal from Aqueous Solutions
Source: Membranes (Basel). 2023 Jul 12;13(7):662. doi: 10.3390/membranes13070662 (PMC10383731; doi:10.3390/membranes13070662)
Supplement: Supplementary file 1 [file membranes-13-00662-s001.zip › membranes-2459058-supplementary.pdf]

*Supplementary Information*

# Development of a Green Polymeric Membrane for Sodium Diclofenac Removal from Aqueous Solutions

**Camila Suliani Raota *, Janaina Silva Crespo, Camila Baldasso and Marcelo Giovanela ***

Área do Conhecimento de Ciências Exatas e Engenharias, Universidade de Caxias do Sul, Rua Franscisco Getúlio Vargas, 1130, Caxias do Sul 95070-560, Brazil
* Correspondence: csraota@ucs.br (C.S.R.); mgiovan1@ucs.br (M.G.)

## 1. Filtration system

The membranes crosslinked according to the design of experiments (DOE) (Table 1) were evaluated through filtration experiments to evaluate permeate flux and sodium diclofenac (DCF) rejection. Filtration experiments were performed in a system illustrated in Figure S1 containing: (A) a feed container (polyethylene, 2 L), (B) a water pump (Shurflo, 1.5 GPM), (C) stainless steel membrane holder in crossflow operation mode (filtration area 12.6 $cm^2$), (D) permeate container (polyethylene, 0.2 L), (E) analog manometer (0.5–14 bar), (F) sphere valve, and polyurethane tubes (10 mm).

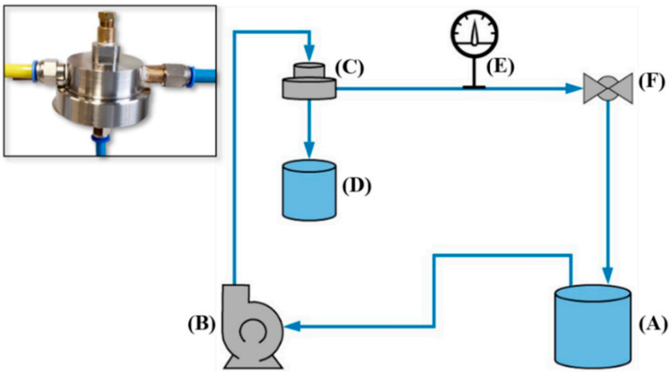


**Figure S1** Graphical illustration of the filtration system: **(A)** feed container, **(B)** pump, **(C)** membrane holder, **(D)** permeate container, **(E)** manometer, **(F)** flow valve. In detail, a photograph of the membrane holder operated in crossflow mode.

## 2. Synthesized membranes

A photograph of the membrane M60_130 is presented in Figure S2. All the other membranes prepared according to the experimental design had a similar yellowish appearance due to the presence of silver nanoparticles (AgNPs) [1], and they were translucid, as seen in the detail of Figure S2.

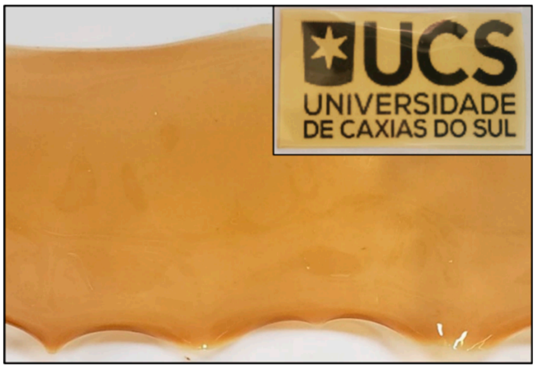


**Figure S2** Photograph of the membrane M60_130. In detail, the translucid characteristic of the membrane.

## 3. Characterization analyses

The crosslinking conditions of time and temperature influenced several properties of the membranes. The following sections present additional results from optical, chemical, and morphological characterizations.

### *3.1. Optical properties using ultraviolet-visible spectroscopy*

The esterification reaction of PVA with citric acid and the effect of crosslinker concentration can be observed in the UV-Vis spectra of Figure S3a. It is possible to observe that pure PVA has almost no absorption in the visible range but absorbs low wavelength UV light (~200 nm), showing two discrete absorption bands at around 285 nm and 335 nm associated with the presence of carbonyl groups (residual acetate groups from PVA synthesis) [2,3]. The presence of citric acid increased the intensity of the band at ~285 nm (esterification reaction), which was even more intense with a higher crosslinker concentration [4]. A comparison of a membrane with satisfactory DCF rejection (M110_110) and another with poor performance (M10_150) is shown in Figure S3b. The overall increase in absorbance (especially in the visible range) may indicate the formation of unsaturation in the polymeric chain [5].

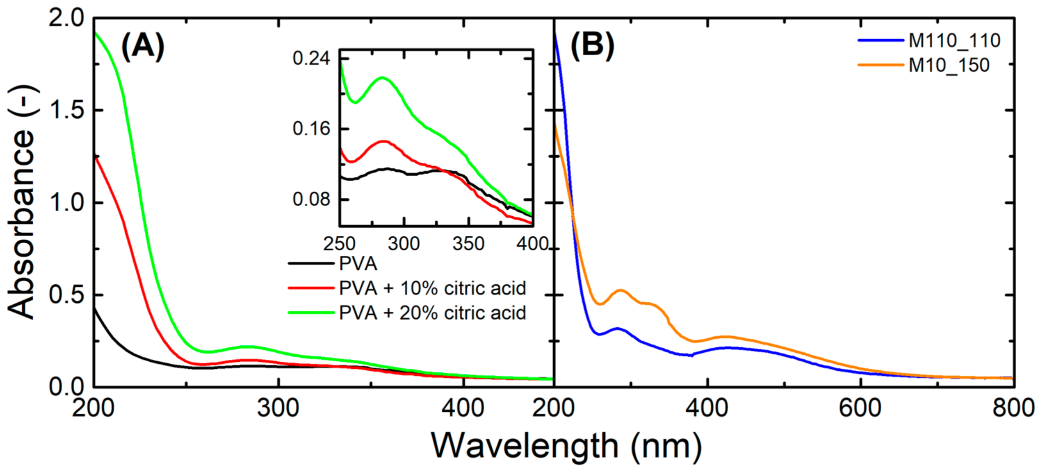


**Figure S3** UV-Vis spectra of **(A)** films made of pure PVA and PVA with citric acid, all submitted to a heat treatment of 110 °C for 110 min, and **(B)** the comparison of spectra of M10_150 and M110_110.

### *3.2. Morphological properties using scanning electron microscopy*

The surface and cross-section images of the membrane M110_110 obtained using scanning electron microscopy (SEM) can be observed in Figure S4. The membrane surface is dense without the presence of visible pores. The areas pointed out by the red arrows in the surface image seem to be impurities derived from the preparation process. The cross-section shows a dense membrane with a thickness of 86 µm. Defects are minor (darker areas), and no visible pores are observable.

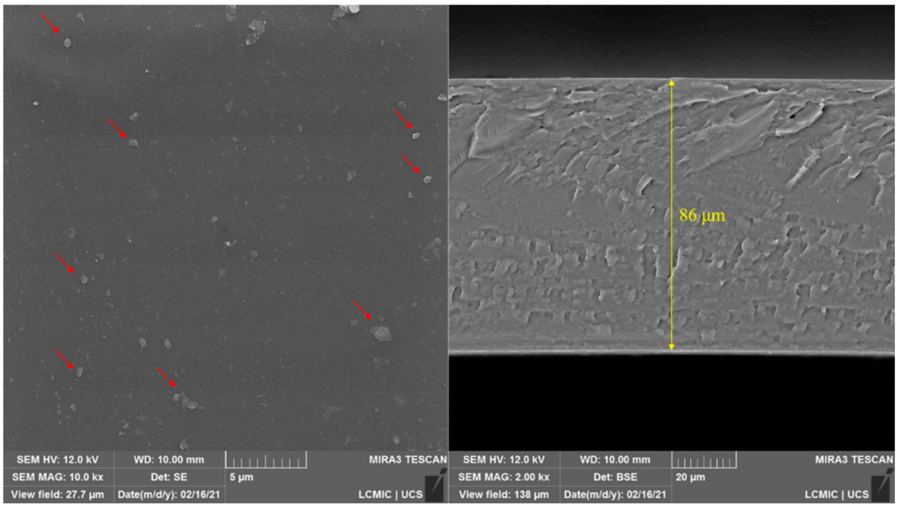


**Figure S4** SEM images of the membrane M110_110 from the surface (right) and cross-section (left). The red arrows point to impurities on the membrane surface, while the measurement in yellow refers to the membrane thickness.

## 4. Statistical results obtained from ANOVA analysis

The results of permeate flux and DCF rejection were statistically analyzed through ANOVA analysis using the software *Statistica 10*. The ANOVA results for the permeate flux and DCF rejection are presented in Table S1.

**Table S1** ANOVA results for permeate flux and DCF rejection obtained using the software *Statistica 10*. Note: F = F-statistics, $p$ = $p$-value. The numbers in red are the results with statistical significance, 95% confidence ($p$ = 0.05)

| Factor [1] | Sum of squares | Degrees of freedom | Mean sum of squares | F | $p$ |
|---|---|---|---|---|---|
| *Permeate flux* | | | | | |
| Time (min) (L) | 15.125 | 1 | 15.125 | 0.3649 | 0.6072 |
| Time (min) (Q) | 1252.2 | 1 | 1252.2 | 30.208 | 0.0315 |
| Temperature (°C) (L) | 7284.1 | 1 | 7284.1 | 175.72 | 0.0056 |
| Temperature (°C) (Q) | 11889 | 1 | 11889 | 286.79 | 0.0035 |
| Time (L) * Temperature (L) | 38935 | 1 | 38935 | 939.25 | 0.0011 |
| Lack of fit | 24104 | 1 | 24104 | 581.49 | 0.0017 |
| Pure error | 82.907 | 2 | 41.453 | - | - |
| Total SS | 104825 | 8 | - | - | - |
| *DCF rejection* | | | | | |
| Time (min) (L) | 392.00 | 1 | 392.00 | 4.3234 | 0.1731 |
| Time (min) (Q) | 16.768 | 1 | 16.768 | 0.1849 | 0.7091 |
| Temperature (°C) (L) | 373.61 | 1 | 373.61 | 4.1206 | 0.1795 |
| Temperature (°C) (Q) | 328.74 | 1 | 328.74 | 3.6257 | 0.1972 |
| Time (L) * Temperature (L) | 96.811 | 1 | 96.811 | 1.0677 | 0.4100 |

| | | | | | |
|---|---|---|---|---|---|
| Lack of fit | 387.59 | 1 | 387.59 | 4.2747 | 0.1746 |
| Pure error | 181.34 | 2 | 90.670 | - | - |
| Total SS | 1883.2 | 8 | - | - | - |

[1] (L) = linear, (Q) = quadratic

**References of the Supplementary Information**


1. Raota, C.S.; Cerbaro, A.F.; Salvador, M.; Delamare, A.P.L.; Echeverrigaray, S.; da Silva Crespo, J.; da Silva, T.B.; Giovanela, M. Green synthesis of silver nanoparticles using an extract of ives cultivar (*vitis labrusca*) pomace: Characterization and application in wastewater disinfection. *Journal of Environmental Chemical Engineering* **2019**, *7*, 103383.
2. Abd El-Kader, F.H.; Gafer, S.A.; Basha, A.F.; Bannan, S.I.; Basha, M.A.F. Thermal and optical properties of gelatin/poly(vinyl alcohol) blends. *Journal of Applied Polymer Science* **2010**, *118*, 413-420.
3. Sau, S.; Pandit, S.; Kundu, S. Crosslinked poly (vinyl alcohol): Structural, optical and mechanical properties. *Surfaces and Interfaces* **2021**, *25*, 101198.
4. Krukowski, S.; Karasiewicz, M.; Kolodziejski, W. Convenient uv-spectrophotometric determination of citrates in aqueous solutions with applications in the pharmaceutical analysis of oral electrolyte formulations. *Journal of Food and Drug Analysis* **2017**, *25*, 717-722.
5. Bolto, B.; Tran, T.; Hoang, M.; Xie, Z. Crosslinked poly(vinyl alcohol) membranes. *Progress in Polymer Science* **2009**, *34*, 969-981.
